# A qualitative study on African immigrant and refugee families’ experiences of accessing primary health care services in Manitoba, Canada: it’s not easy!

**DOI:** 10.1186/s12939-016-0510-x

**Published:** 2017-01-09

**Authors:** Roberta Lynn Woodgate, David Shiyokha Busolo, Maryanne Crockett, Ruth Anne Dean, Miriam R. Amaladas, Pierre J. Plourde

**Affiliations:** 1 Rady Faculty of Health Sciences, College of Nursing, University of Manitoba, Winnipeg, MB R3T 2N2 Canada; 2 Departments of Pediatrics and Child Health, Rady Faculty of Health Sciences, Max Rady College of Medicine, Medical Microbiology and Community Health Sciences, University of Manitoba, Winnipeg, MB R3E 3P5 Canada; 3 Nor-West Co-op Access Center, 785 Keewatin Street, Winnipeg, MB Canada; 4 Medical Officer of Health, Winnipeg Regional Health Authority, 490 Hargrave Street, Winnipeg, MB R3A 0X7 Canada

**Keywords:** Immigrant, Refugee, Primary care, Health services’ access and use, Social determinants of health, Qualitative research

## Abstract

**Background:**

Immigrant and refugee families form a growing proportion of the Canadian population and experience barriers in accessing primary health care services. The aim of this study was to examine the experiences of access to primary health care by African immigrant and refugee families.

**Methods:**

Eighty-three families originating from 15 African countries took part in multiple open ended interviews in western Canada. Qualitative data was collected in six different languages between 2013 and 2015. Data analysis involved delineating units of meaning from the data, clustering units of meaning to form thematic statements, and extracting themes.

**Results:**

African immigrant and refugee families experienced challenges in their quest to access primary health care that were represented by three themes: *Expectations not quite met*, *facing a new life, and let’s buddy up to improve access.* On the theme of *expectations not quite met*, families struggled to understand and become familiar with a new health system that presented with a number of barriers including lengthy wait times, a shortage of health care providers, high cost of medication and non-basic health care, and less than ideal care. On the theme of *facing a new life*, immigrant and refugee families talked of the difficulties of getting used to their new and unfamiliar environments and the barriers that impact their access to health care services. They talked of challenges related to transportation, weather, employment, language and cultural differences, and lack of social support in their quest to access health care services. Additionally, families expressed their lack of social support in accessing care. Privately sponsored families and families with children experienced even less social support. Importantly, in the theme of *let’s buddy up to improve access*, families recommended utilizing networking approaches to engage and improve their access to primary health care services.

**Conclusions:**

African immigrant and refugee families experience barriers to accessing primary health care. To improve access, culturally relevant programs, collaborative networking approaches, and policies that focus on addressing social determinants of health are needed.

## Background

Immigrants and refugees form a considerable and growing proportion of the Canadian population and their access to health care services is increasingly a priority. Between 2006 and 2011, 1,162,900 foreign-born nationals made up 17.2% of the foreign-born population and 3.5% of the total population in Canada [1]. In 2010, 12.5% (145,700) of all immigrants arrived from Africa particularly from Algeria, Morocco, and Nigeria [1]. After settling in Canada, immigrants and refugees often experience a decline in their health status yet we know very little about African immigrants’ and refugees’ experiences of accessing health care services [2, 3]. Access to health care involves a fit between the client and health care system where the client identifies health care needs, looks for health care services, reaches out, acquires, and fulfils the need for these services [4, 5]. Health care services are expected to be approachable, acceptable, available, accommodative, affordable, and appropriate [5].

Immigrants and refugees come to Canada in different immigration categories that include convention refugees (privately sponsored or government sponsored), asylum seekers, economic class, and family class immigrants. Convention refugees are people who flee from their home country for reasons that include fear of persecution [6] while asylum seekers are people awaiting determination of their requests for refuge [7]. Privately sponsored refugees are either convention refugees or asylum seekers who are referred for resettlement by a sponsor in Canada who commits to support them for 1 year [8]. Government assisted refugees are convention refugees referred for resettlement by a referral organization (e.g. the United Nations Refugee Agency (UNHCR). Their resettlement is supported by the Canadian government or the province of Quebec (for up to 1 year) [8]. Economic class immigrants are people who come to Canada for non-humanitarian reasons under immigration classes like federal skilled worker class, provincial nominee class, the Canadian experience class, or family class where they are sponsored by an immediate family member [9].

Immigrants often present with a favorable health status on arrival but over time develop the same or even worse health status than Canadian-born populations. Refugees on the other hand often arrive in poorer general health status than their immigrant counterparts [10] and often present with ill physical and mental health status [11]. Contributing to their likelihood for poor health is a difficult migration journey that may include migration from countries experiencing violent conflicts, forced migration at short notice, and living in refugee camps, along with unfavorable social determinants of health [3, 12–18].

Although immigrants and refugees may present with increased health care needs, they face considerable barriers to accessing health care services in Canada [11, 19–23]. Immigrants experience poor patient-provider communication due to language barriers, nonprofessional interpreters, physicians being too busy to listen or lacking empathy with their health concerns, inadequate referral to specialized care resources, inadequate social support, differences in expectations, and lack of culturally sensitive care [24, 25]. Others are challenged by socio-cultural differences and economic difficulties [26].

Like immigrants, refugees experience similar problems. For example, a qualitative study that involved convention refugees (*n* = 27) and refugee claimant (*n* = 28) mothers revealed that the mothers had difficulties accessing care when needed. The difficulties were due to income level, health insurance problems, lack of knowledge about services, and feelings of being judged as a parent [27]. Additionally, one third of mothers witnessed or were the object of racism and discrimination in the health-care system [27]. In another study that focused on health care providers’ perspectives of refugees access to health care services [28], refugees experienced additional barriers in language and interpretation, differences in culture, health care coverage, availability of services, isolation, poverty, and poor transport. In a dimensional analysis of refugees access to health care, it was found that a sense of discrimination and stigmatization, and logistical concerns were additional barriers to refugees’ access to health care services [29]. Moreover, refugees fear accessing health care services where their immigration status can be questioned or reported to law enforcement authorities [11, 27, 28] and being denied care [21]. Similarly, asylum seekers found it difficult to access care because it was too expensive, were suspicious of the health care system, and felt the health care professionals had unfavorable attitudes towards them [11].

While there is a growing body of literature specific to immigrants’ and refugees’ experiences in accessing health care, little is known about the experiences of African immigrant and refugee families. In two systematic reviews on immigrants’ experiences of accessing primary care and maternity services in Canada [24, 25], none of the 33 studies (27 qualitative, 5 quantitative surveys, and 1 mixed-methods) focused on African immigrant and refugee families. South Asian and South-East Asian immigrant populations were the focus of most of the studies. Accordingly, better representation of other racialized groups, more information-rich data including immigrant participants’ voice, more work on the perceptions and experiences of parents raising children, and a broader focus on primary health care were recommended [24]. In addition, more information on experiences based on the type of immigrants (e.g. family sponsored immigrants) is warranted.

Research that has investigated immigrant and refugee families’ experiences of accessing primary health care services in Canada has mainly been national surveys [30] and focused on non-African immigrant and refugee populations [24–26, 28, 31–33], women [25], mothers with ill children [23, 27, 33], or only African women [34]. The challenge of overly relying on national surveys and focusing on participants other than the African immigrant and refugee families themselves excludes an important piece of the social construction of their experiences. Additionally, experiences from African men within the family are a critical and under researched piece in the literature. African immigrants and refugees continue to make up a growing proportion of the Canadian population. Between 2006 and 2011 Africans made up 12.5% of newcomers to Canada which was an increase from 10.6% in the preceding 5 years [1]. Therefore, understanding their experiences and opportunities for better access to health care services is important. Accordingly, we undertook a qualitative study where we explored experiences of access to primary health care by African immigrant and refugee families.

## Methods

We utilized a qualitative research study design to examine African immigrant and refugee families’ experiences of accessing primary health care services. This approach allowed families to present their experiences based on their own reality.

### Participants and data collection

Eighty-three families that had recently (≤6 years) immigrated to a city in Manitoba took part in the study. In this manuscript, we focus on views of parents from the families. The process of data collection took place at the preferred location (e.g., home, church) of the families and was facilitated by trained research assistants who worked under the supervision of RLW. Data was collected in Canada during a time when the federal government had passed legislation that limited access to the Interim Federal Health Program (IFHP). Families were recruited through purposive sampling. Parents were engaged in one to three individual open-ended interviews for a total of 141 interviews in a language of their choice. Each interview lasted between 60 and 180 min long and took place at locations that were most comfortable for participants. Field notes were recorded after each interview to describe the interview context (e.g., families’ non-verbal behaviours, communication processes). Overall, 44 parents chose to speak in English, 15 in Kiswahili, 12 in French, 17 in Amharic, three in Arabic, 16 in Somali, and one in Bari. Certified translators were trained and made available to provide translation and back interpretation of those interviews not conducted in English. Allowing parents to communicate in languages they were most comfortable with, helped to ensure rigor in a cross language research study [35]. Parents were asked to share experiences of accessing health care services for themselves and their families. In the interviews we asked questions like “please describe what it was like to go to your first health encounter,” “please describe what it was like to go to your child’s first health encounter,” and “how did this experience make you feel?”

### Data analysis

All interviews were digitally recorded and transcribed verbatim. In keeping with the qualitative paradigm, data collection occurred concurrently with data analysis. All data emerging from interviews and field notes informed data analysis with careful line-by-line analysis of the transcripts, and iterative analysis [36, 37]. Data analysis involved delineating units of meaning from the data, clustering units of meaning to form thematic statements, and extracting themes. Demographic data was analyzed using SPSS (Statistical Package for the Social Sciences) version 22.0 [38]. Reflexivity was maintained through on-going documentation in an audit trail and comparing this reflection against the data in the analysis process [39]. Through prolonged engagement in the topic area, the researchers engaged in further reflection and development of the findings.

## Results

Our results are presented as follows: after summarizing the characteristics of the participants, we describe their challenges in their quest to access primary health care services.

### Participant characteristics

Of the 108 parents that took part in this study, there were 70 (65%) females and 38 (35%) males between the ages 23 and 74 years (Mean = 41.2 SD = 10.6). Fifty parents preferred to take part in the interviews as a couple (*n* = 25 couples), with the rest taking part in individual interviews. Eighty-three (77%) of the participants were married, four (4%) were single (never married), three (3%) were separated, six (6%) were divorced, and one (1%) participant did not indicate her marital status while 11 (10%) were widowed. Participants were originally from Burundi (4%), Cameroon (1%), Congo-Brazzaville (6%), the Democratic Republic of Congo (DRC) (22%), Eritrea (4%), Ethiopia (19%), Ivory Coast (4%), Liberia (1%), Mali (1%), Rwanda (2%), Sierra Leone (3%), Somali (18%), South Sudan (5%), Sudan (10%), Uganda (1%), and Zimbabwe (1%). Participants had resided in Canada for about 4 years and had approximately four people in their household. Twenty-eight (26%) participants immigrated to Canada as family class immigrants, 68 (63%) came as Government Assisted Refugees, and five (5%) as economic class immigrants while seven (6%) came as other (e.g. Asylum seekers). Among 102 participants that revealed their level of education, eight (8%) had a graduate level of education, 24 (24%) had less than high school education, 41 (40%) had a high school diploma, and 29 (28%) were attending some post-secondary institution. Eighty participants provided their income information. Of these participants, 72 (90%) identified themselves as low income earners with only 50 (46%) reporting employment. More than half of the participants were Christians, while 35 (32%) were Muslims.

### The experiences of African immigrant and refugee families access to primary health care services

African immigrant and refugee families presented with various health conditions that reflected different health needs and a possible decline in their health status. Some families arrived in Canada after a difficult immigration journey that included experiencing violent conflicts and living in refugee camps. Life in a refugee camp was portrayed as very difficult. Upon arriving in Canada, families lived under unacceptable conditions (e.g. poor housing) and were challenged in their quest to access health care services. Families shared their experiences on accessing health care services that touched on the social determinants of health including health system and services, physical environments, employment and job security, stress and illness, culture, language, immigration, and social supports. Their narrations were summed up in three themes of *expectations not quite met*, *facing a new life,* and *let’s buddy up to improve access*. The first two themes reveal the barriers and gaps to accessing care by different categories of immigrant and refugee families as highlighted in Table 1. The last theme describes immigrant and refugee families’ recommendations to improving access.

**Table 1:** Problems unique to the major types of immigrants and refugees

Family class immigrants	Government assisted refugees	Privately sponsored refugees
• Unmet expectations • High cost of medication and non-basic health care services. Could receive support from supporting family.	• Unmet expectations • High cost of medication and non-basic health care services. Barriers are greater for low income families.	• Unmet expectations • High cost of medication and non-basic health care services. Barriers are greater for low income families.
• Less than ideal care that lacked cultural sensitivity	• Less than ideal care that lacked cultural sensitivity	• Less than ideal care that lacked cultural sensitivity
• Unfamiliarity with a new system of health. (Dependent on host family for orientation)	• Unfamiliarity with a new system of health. Could benefit from government funded orientation about the health care system	• Unfamiliarity with a new system of health. Dependent on their sponsor for orientation about the health care system
• A lack of transport services. Could receive support from the supporting family	• A lack of transport services especially for low income families.	• A lack of transport services especially for low income families.
• Employment challenges. Challenges are greater for families with children	• Employment challenges. Could benefit from government funded employment assistance services	• Employment challenges. Challenges are greater for families with children
• Language and linguistic challenges	• Language and linguistic challenges	• Language and linguistic challenges
• Lack of access to interpretation services. (Dependent on host family for interpretation)	• Dissatisfaction with translation services	• Lack of access to interpretation services. (Dependent on the type of sponsor, i.e. organization or individual)
• Limited social support. (Dependent on host availability)	___________	• Limited social support that includes orientation. (Dependent on the type of sponsor, i.e. organization or individual)

### Expectations not quite met

On the theme of ‘expectations not quite met’ immigrant and refugee families presented with high expectations of access to health care than they experienced. Families had grown up in African countries and were used to a certain level of access to health care. As such, families expected to receive similar or even better access to health care. However, their expectations were not met because of health system barriers. For instance, in some African countries, one gets prompt access to care while in Canada sometimes getting access to care can be a “challenge.” Such immigrant and refugee families perhaps expected access to care to be prompt. When their expectations were not met, they were disappointed.

*In Africa when you go to the hospital, they treat you quickly and … if the medication is finished they give you, you go back and collect it… But here they said you have to wait, you have to see your doctor first, and the doctor says that to see a dermatologist is not easy … you see that is a challenge. In Africa you go and they treat you, you have your medicine directly, see we’re used to that. But here in Canada you have to wait … you see that is a challenge.* (Sierra Leone Female, Government Assisted Refugee)

When families in this study went to health care service points, they expected primary health care services to be at least available, at an affordable cost, and acceptable. However, this was not the case. Both immigrant and refugee families were disappointed, distraught, and struggled to understand lengthy wait times at health care service points, high cost of medication and non-basic care. Barriers to accessing health care services were similar to those presented in the quote below.

*We took the boy to Emergency and…waited…until 3:30 am. So we took him at 8:00 pm and then we waited until 3 a.m. and nothing happened and so we were told this doctor is off duty and another doctor will come, but no one showed up so we were so tired at the end we just brought him home.* (Ethiopian Male, Privately Sponsored Refugee)

In spite of the disappointment, some families made sense of the lengthy wait times by believing there was a “*shortage of health care providers”* and therefore, the reason why “it took *a long time for people to get the treatment they wanted”* (Ethiopian Female, Government Assisted Refugee). Expectedly, they made recommendations to hire more physicians.

Although immigrants and refugees had obtained the provincial health insurance coverage, the health insurance did not cover for medication and non-essential care. The cost of medications and non-essential care (e.g. dental care) was very high. Meeting these costs was challenging particularly for low income immigrant and refugee families that could barely meet their monthly bills. Families, especially those with children, were particularly helpless because the costs of the health care services were too high for them to address any of their health needs.

*Most importantly I need dental health care for my children…which is expensive for newcomers whose families have a low income and the government does not cover that. You know, we have to pay for dental health care and we can’t, so it is very difficult for me and for other people who have low income like me too. So second of all if you have an eye problem which is expensive, low income families can’t pay for the medication or eyeglasses, you know it’s very difficult for families with low income.* (Ethiopian Female, Government Assisted Refugee)

Immigrant and refugee families also identified certain experiences of care which they perceived as inappropriate, perhaps unacceptable and could stop them from accessing care. The quality of health care provided was less than ideal. One of the participants who shared experiences of care mentioned that Africans may expect to be given medication following their interaction with the care provider and, if not provided, may be upset and perhaps not seek care in future.

*I think the other problem maybe you have to correct me because of our kind of African mentality about medicine, whenever you go to the hospital in Africa there at least they give you some, some medication and here sometimes they ask you to go and drink some water and that makes them very, very upset. (*South Sudanese Male, Privately Sponsored Refugee).

Another participant echoed the missed expectations of ideal care. He mentioned that families especially those with children expect someone to attend to them when they get to the hospital. When no one seems to care, they can get “agitated.”

*I mean for a parent to see a child that is sick and then you just sit and you’re in the hospital that is supposed to be taking care, and the child is not taken care of, of course you become angry, you know a little bit agitated.* (Sudanese Male, Government Assisted Refugee)

Others shared experiences where they felt the care provider did not perform their role as expected.

*Recently I was sick and I went to see a doctor and I was told I have a blood infection, so the doctor didn’t even bother to examine me or look at me closely, she just gave me a prescription…so they don’t even bother to check if anything, if what they give you is really appropriate or whether it works. So I don’t think I would want to see a doctor or go to seek health service.* (Ethiopian Female, Privately Sponsored Refugee).

### Facing a new life

On the theme of ‘facing a new life,’ immigrant and refugee families talked of the difficulties of getting used to their new and unfamiliar environments and the barriers that impact their quest to access health care services. They talked of challenges related to transportation, weather, employment, language and cultural differences, and lack of social support in their quest to access health care services. With regard to challenges in transportation, families could not reach out for care because they were new to the city and relied on public buses that only operated on certain schedules and on specific routes. As such participants needed an alternative form of transport (e.g. a car) which they did not have.

*I don’t have a car and I don’t drive, so sometimes going to my appointments it’s hard because I, I do want to go for these appointments, but when it’s snowing outside and I don’t drive, I don’t have a car, it makes it difficult for me to make it a point to go to all of my appointments*. (Somali Female, Privately sponsored Refugee)

During winter months, families faced additional challenges of the cold weather where they needed winter clothing. For those who tried to access care, it was especially difficult when the family had children.

*We had these small kids, we didn’t have even a nice stroller, we didn’t have a stroller; we didn’t have even good clothes, like winter clothes…my wife was pregnant, which was really heavy for her already. Oh my God, we didn’t even know how to use the bus properly…so (chuckle) because we were living in N [name of location], we had to go to S [name of another location in the city] to meet with a doctor to check up my wife. And (chuckle) it was horrible.* (DRC Male, Government Assisted Refugee)

In addition, families found it challenging to access care because of unemployment or underemployment. When unemployed or underemployed, families could not benefit from health insurance coverage provided by their employers. They had to buy health insurance which was expensive. The families continued to look for full-time employment which could come with health benefits. Also a lack of employment impacted families’ housing conditions. Often, immigrant and refugee families lived in cheap houses that were in a bad state. Such poor housing conditions affected their health and increased their need to acquire health care services.

*I’m concerned about my kid, my daughter she have a tooth problem. I have to buy insurance but how can I? I don’t have money now, I don’t have full-time job now. You know I’m looking for a job, a full-time job to get some you know some full benefit, I’m trying to do that but I can’t, I can’t.* (Ethiopian Female, Government Assisted Refugee)

*I don’t know, because of my son. When we were first living at K [location of their residence]…it was not a very clean place to live there, so that’s where he started having his breathing problems.* (Somali Female, Government Assisted Refugee)

Dealing with the barriers caused or shaped their health status with participants reporting increased stress and/or illness as illustrated by the following

*So it was very hard for us coming here with the family and we had health problems and it wasn’t easy to get medical help starting from understanding the language and waiting to get a family doctor took nine months. We had to go to clinics, walk-in clinics where it was hard to make the appointment, the weather was hard, the children get sick because of the changes, so it was very difficult for us.* (Ethiopian Female, Government Assisted Refugee)

In addition to the barriers discussed, the language and cultural differences and a lack of social support really emerged as key barriers impacting access to health care services. As such, we present them as sub-themes.

#### Linguistic and cultural differences

Linguistic and cultural differences made it difficult for study participants to engage in health care. African immigrant and refugee families found it difficult to communicate and understand a Westernized concept of health, health care systems, and health care approaches. As a result, participants felt misunderstood by care providers which made them frustrated.

*So once I was there at the hospital and I didn’t know what to say or how to go on about it. To think about it, even now is too stressful. I was thirsty and I wanted water and I didn’t know how to say, “please give me water.” So they were really concerned because the baby was already half out and I had this pain at my back and I still have a pain on my back and they came and said “is there anything we can do for you now?” I was just looking at them cause I didn’t know what to say to them.* (Ethiopian Female, Government Assisted Refugee)

Some families found it challenging to access care because of language problems. Health care service points were difficult to locate, especially when English comprehension was poor.

*The doctors’ offices were made like very complicated to be accessed…is a little bit tough… I remember I went for CT scan and general check-up; it took me time to locate myself and to look for where I can get this service. So it is accessible actually but for those who are with the language problem it is very challenging.* (South Sudanese couple, Privately Sponsored Refugees)

In other situations, it was difficult to adequately express symptoms and comprehend the health care provider’s conclusions. Some health conditions did not have appropriate English translations, thus wrong information was sometimes conveyed. Failing to capture the correct translation sometimes made immigrants and refugees question the diagnosis and treatment plan which created tension between them and their health care providers. Failing to understand the physician also resulted in some immigrants and refugees to not follow the treatment plan or even walk away from the doctor’s office without a treatment plan.

*Yeah, for the immigrants the main thing is the language, its, that’s what the difficult thing. When you understand it, it makes it easier but sometimes you don’t understand but you just say “okay” and just leave, but you have no idea what, what they told you*. (Ethiopian Male, Family Sponsored Immigrant)

*Last night my husband was away at work and one of my daughters was really sick, she has gooryaan (pinworms) and I took her to the emergency and I wanted to tell them what’s wrong with her, but they couldn’t understand me, I couldn’t really explain…so that language is really difficult sometimes to just get your message through to the next person.* (Ethiopian couple, Government Assisted Refugees)

Other families found it challenging to understand certain health care procedures and felt that health care providers were not interested in helping them understand. One female refugee shared her experiences at the maternity unit where she appeared to have limited understanding of the fetal monitoring procedure. The health care provider did not appear to educate her.

*You know we went to the fetal monitoring machine to check the child’s heart, the nurse tied me on my stomach, she did not talk to me, and she did not help me. I said I need stand, she said “No.” She continued to write something on paper…I said “Excuse me please can you help me?” My husband said, “If you cannot help then untie her pleas*e.” (Ethiopian Female, Government Assisted Refugee)

Differences in cultures also resulted in families experiencing frustration in the health services.

*When I remember it…I become emotional and I cry, so for three days without eating and without drinking, I was in hospital. I gave birth and I didn’t eat or drink. Not because they didn’t offer me food but the food they gave me was foreign and I didn’t know how to eat it or how it will be like and my husband cannot bring me food because there is no one to leave the children with.* (Ethiopian Female, Government Assisted Refugee)

Immigrants and refugees also identified certain aspects of care to be culturally inappropriate.

*When I was getting my treatment I used to really feel shy that I have to undress in front of a male doctor because in my culture it prohibits me from doing that with, if I can’t find a female physician…because if he’s a male, I used to feel very uncomfortable that I had to undress and sometimes I have to show him my private parts and even though this is for my treatment but it used to make me feel very uncomfortable, and I’m still not comfortable*. (Somalian Female, Privately Sponsored Refugee)

In spite of families’ inability to communicate appropriately to their care providers, there were very few multilingual health care providers that attended to African families. The health care system relied on interpreters to improve communication between the immigrant and refugee families and their care providers. However, it appeared that using interpreters was not always a solution.

*It’s like there’s only one doctor who speaks the language and he has so many patients because of the language, it would be nice if there was a nurse or someone who speaks the language because you know everything is confidential.* (Ethiopian couple, Privately Sponsored Refugees)

Where the government made efforts to provide interpretation services, the services were mainly directed at government assisted refugees. Other refugees (e.g., privately sponsored refugees) could not use interpretation services, leaving them to feel alienated and expressing the need for help.

*“Name of Agency” provides people who are sponsored by the government with an interpreter when they go to see a doctor, but privately sponsored people will have difficulties. So it would be nice if it is possible like if someone can accompany them someone who can speak their own language when they want to see a doctor.* (Ethiopian couple, Privately Sponsored Refugees)

#### You are on your own!

On the sub-theme of “you are on your own” immigrant and refugee families went beyond the aforementioned challenges to express how difficult it was to access care because they lacked social support. In our study, 22% of the participants were separated, divorced, or widowed and faced greater challenges because help was limited. Similarly, family class immigrants appeared to have limited support. Government assisted refugees were more likely to benefit from government funded social programs but the level of support for families with children was inadequate.

*It would have been nice, she [support worker for families of children with disabilities] comes from an agency focusing on disabilities and it would be nice if they could extend the hours and she could come and help us a bit longer than what she’s doing for us right now that is one area that we’re facing trouble with.* (Somali Couple, Family Class Immigrant)

Another family whose son had a physical disability talked about making progress but reflected on how they struggled in the preceding years.

*Health wise our son, we are not there yet and it is very challenging…so I was by myself to care of my son, challenging health-wise…because my son has a disability, hearing disability, so it was kind of tough as newcomer. Yeah those people are struggling to survive and having a disabled son is very, very challenging and yes I was trying to work hard, study hard to overcome it, and making hope for a better but it was so challenging.* (Eritrean couple, Convention Refugee)

Because families lacked support, they did not engage in health promotion practices as they could have desired.

*Generally like the way of life being so busy with raising a young family is a little heavier on us to do…so us being busy with work and raising children makes us to neglect the preventions like doing more exercise, strengthening the body…any issues affecting the body…we focus more caring for the children, work…if you want to go to gymnasium or health facilities, you’ll go with the kids and more often instead you can’t do much.* (Ethiopian couple, Privately Sponsored Refugee)

When support was available, it was mainly directed at government assisted refugees.

*I didn’t have a language problem because I was sponsored by the government…the government paid for my interpreter, but I had a friend, for example she was privately sponsored and she asked if the person who interpret for me if he could help her out, but he said you know I won’t get paid*. (Ethiopian Female, Government Assisted Refugee)

Privately sponsored refugees had to rely on the individual(s) who sponsored them, which at times was not always ideal.

*When you are sponsored by the government…you’ll be given someone to accompany you to see a doctor…they will guide you on what you need to do. But when you are sponsored by a private person that person has all those responsibilities to do for you but that person might not have the time to take you around to show you places, to accompany you to each doctor’s visi*t. (Ethiopian couple, Privately Sponsored Refugees)

### Let’s buddy up to improve access

In the theme of “let’s buddy up to improve access,” families acknowledged that it was challenging for immigrants and refugees to access health care services in Canada. They went beyond the aforementioned challenging experiences. They made recommendations for using collaborative networking approaches and developing culturally relevant programs to improve access to health care. Families made suggestions of tailoring services to address the specific needs of African immigrant and refugee families. Tailored services could include special clinics and health education and awareness forums for the African immigrant and refugee population.

*There should be kind of a special clinic for newcomers that will help out. The second thing is like there should be some kind of awareness or information sharing in the communities, seminars in the communities telling about the health system, how to access a doctor.* (Egyptian male, Privately Sponsored Immigrant)

Apart from tailoring existing programs, immigrant and refugee families suggested tapping into social networks as a way of improving access to health care services. There is a growing proportion of African immigrant and refugee families that have lived in the country for some time. These families could be familiar and be best placed to understand the challenges experienced by newcomer immigrant and refugee families in their quest to access health care services. As such, African and other like-minded families could form networks to support new immigrant and refugee families. Some of our study participants shared experiences of getting help to access services as well as offering to assist others to access health care services.

*My husband made the first appointment. He met someone at the mosque who knew a family doctor that went to the same mosque. That person introduced my husband to the doctor at the mosque and my husband asked “Could you also be the family doctor for my children?” and the doctor told them about how they could go to the clinic and make the request at the clinic and since then we’ve been taking them there.* (Somalian Female, Privately Sponsored Refugee)

*We just came and our son was very sick and he was throwing up and he had fever, so we just came and we didn’t even get a chance to go and get a health card, so he was sick and our friend “M” he made an appointment and he took us to a walk-in clinic on one of the local streets. And uh we, our friend had to pay 55 dollars to get a service for our son and it was a walk-in clinic at one of the local streets*. (Ethiopian couple, Privately Sponsored Refugees)

Immigrants who resided in the city for a longer time shared experiences of reaching out to support new families who had to access health care services. They volunteered their time and resources to support the new families.

*We do everything. We tell them where to go, you know when the child is sick we tell them personally I’ll be taking kids for other people and I say well you don’t have a vehicle, you are new here, but I’ll come, and my wife was doing the same thing…*

(South Sudanese male, Family class Immigrant)

## Discussion

This study examined African immigrant and refugee families’ experiences of accessing health care services in Canada which adds to an area that is critically missing: African families’ experiences [24]. While national surveys [30] and qualitative studies have focused on other ethno cultural populations [22–26, 31–33], or only on African Canadian women [34], our study adds more qualitative evidence on African families’ experiences. It emerged from their deliberations that they were experiencing difficulties in accessing health care services.

The families in this study presented with poor social determinants of health that included low income and social status, poor levels of education and literacy, and living in inadequate physical and social environments. The families with an average of four people reported annual incomes below poverty indices with only 45% of them claiming to be employed. Living under these conditions could contribute to a decline in health [40] and the need for better access to health care services. Unlike other low socioeconomic class Canadians, immigrant and refugee families face additional challenges of being new to the country. Some arrive in Canada after experiencing a difficult migration journey that may include living in refugee camps and experiencing violent conflicts [3, 12–18]. As newcomers, immigrant and refugee families often have to start life from nothing and find themselves at disadvantaged social positions on matters such as race/ethnicity, gender and socioeconomic status. Often times, immigrants find themselves in ambiguous and hostile relationships with the country and its institutions including health services [41].

Expectedly, families were in search of ways to address their social determinants of health (including access to health care services) and stressed upon the need for others (e.g. the government and health care providers) to play a more proactive role in addressing these determinants. Their desires were accentuated at the time of data collection because the federal government had passed legislation limiting access to the Interim Federal Health Program (IFHP) [42]. IFHP provides health insurance for medication, emergency dental and vision care, translation, and prostheses for protected persons who include resettled refugees, and refugee claimants [43]. Some of the shared experiences could be as a result of the legislative changes which resulted in decreased benefits and coverage for some immigrant categories that included privately sponsored refugees and claimants from countries that do not normally produce refugee claimants. Presently, immigrants and refugee families are eligible for certain provincial drug and dental care programs (e.g. pharma care) if they demonstrate that their income is seriously affected by the cost of prescription medication. Immigrant and refugee families with a low income and who reside in certain parts of the city are also eligible for a few low cost dental programs. While these programs are beneficial, they are few and limited. To improve access to care, we recommend better awareness of existing programs, and more provincially funded dental programs in other neighborhoods where more immigrant and refugee families reside.

Families expressed frustrations at accessing health care services because their expectations of the health care system were not quite met and because they had to face a new way of living. Encountering unmet expectations and a new way of life created barriers in their access to health care. Similarly, in another Canadian study, immigrants found it difficult to access care because of geographic and economic barriers [26] while asylum seekers were limited by lack of awareness of the health systems [11]. Unlike findings in these studies [11, 26], we found that families were challenged by lengthy wait times at health care service points, high cost of medication and non-essential care, and inadequate child care support. Our findings support the work by Levesque, Harris and Russell [5] who suggest that access to care is affected by providers, organizations, institutions and systems barriers. In the short term, we suggest that resources be harnessed to reduce such health systems barriers while in the long term, we recommend that policies and programs focus on eliminating these barriers (e.g. through increasing child support services for newcomers with children), increasing the number of culturally competent health care providers [26], and subsidizing indirect and non-basic health care costs for low income newcomer families. With regard to facing a new life, Levesque, Harris and Russell [5] mention that access to health care is limited by populations, communities, households and individual barriers. Similarly, we found that families were challenged by living in a new environment where they faced a lack of transport, poor weather conditions, employment challenges, linguistic and cultural differences, and lack of social support. In our study, linguistic and cultural and lack of social support were the most reported barriers of in their new life.

Regarding linguistic and cultural barriers, families were frustrated with being unable to communicate effectively with care providers. Similarly, in a meta-ethnography on immigrant women’s experiences of maternity services in Canada, it was reported that communication was a barrier in accessing care [25]. Health care providers and patients expected the other to do more to overcome the barrier [25]. The families in our study desired care that was appropriately translated and culturally relevant. Therefore, to improve access, we make suggestions for more culturally accommodative health care services where care providers reflect upon their cultural values, beliefs, and practices and those of others. Approaches where care providers are more aware of their communication skills and those of others and work towards modifying their care skills to accommodate African immigrants and refugees could be beneficial [44]. We believes culturally competent care could be developed through networks between health care and social service providers and newcomer communities [45]. To reduce linguistic barriers, information sharing opportunities could be developed where educational materials that focus on accessing health care services could be presented to new African families in multiple languages.

Immigrants and refugees have a high risk of developing physical and mental health conditions because they are separated from some of their cultural norms, values, and social support systems [46, 47]. On the sub-theme of “you are on your own!” it was evident that families, especially those with limited connections and or built relations in the city, desired to be supported. They needed help in accessing health care services, taking care of sick children, or in health promotion practices. Similarly, immigrant women that sought to utilize maternity services in Canada experienced weak, inadequate social support services [25]. Mothers experienced separation from their social networks, and had poor access to child care services [25]. Such lack of adequate social support can lead to mental health problems like postpartum depression. Importantly, both immigrant and refugee families in our study made recommendations of networking and partnering with health care providers to improve access to health care services.

On the theme of “let’s buddy up to improve access,” our study participants shared experiences of offering and receiving support to access health care services. We welcome the suggestion to offer support but recommend that the level of support stops short of providing translation at the health care settings. We believe African immigrant and refugee patients’ confidentiality particularly at the health care settings needs to be respected. Study participants also made suggestions of tapping into existing social networks e.g. community groups, friends, and relatives to improve access. We believe African community organizations in the city could act as knowledge broker organizations that could link service providers with immigrant communities [19, 48]. Knowledge broker organizations and social networks can be useful at developing connections between network members for supporting immigrants to access services and develop cultural competence within the health care system [48, 49]. The research team members believe the development of mentorship programs would especially be of value. African immigrant and refugee families that are new to Canada could be linked up with African families that have lived in the country for some time.

From study findings and analysis, the research team recommends a plan that targets health care and social service providers, community members, and policy makers (see Table 2). Such a plan could focus on improving African immigrant and refugee families’ understanding of health and the health care system, working with translators, and the process of accessing care.

**Table 2:** Networking plan to improve African immigrant and refugee families’ access to health care services

Immigrant/refugee families’ barriers	Health care and social service providers’ actions	Community members’ actions	Policy implications
• Unmet expectations - Lengthy wait times - shortage of health care providers - high cost of medication and non-basic health care - less than ideal care	• Provide more walk in clinics in sections of the city where recent African immigrant and refugee families live • Provide more continuous education forums that focus on providing health care to immigrant and refugee populations	• Engagement of key community leaders on developing a buddy support system to help immigrant and refugee families access health care services • Training and remuneration of key community leaders who support immigrant and refugee families access health care services	• Provision of resources specifically for newcomer families • Subsidising certain non-essential health care costs (e.g. dental care) as well as medications for newcomer families who do not have health insurance
		• Community members to liaise with health care providers and social service providers (e.g. schools) to connect with new immigrant and refugee families	• Fast track the recruitment and retention of internationally trained health care professionals
• Facing a new life: - Language and cultural differences	• Health care providers need to be aware and supportive of linguistic needs and cultural differences of the immigrant/refugee families	• Community members to link newcomer families from similar linguistic and cultural backgrounds to health care services	• Provide and/or increase funding to networking organizations that facilitate immigrant and refugee families’ access to health
- Getting used to a new and unfamiliar environment	• Working as a team, health care and social service providers need to promote awareness and information sharing sessions to immigrant and refugee communities on health, health care systems, and accessing health care services	• Community members to liaise with health care providers to offer workshops and training sessions to immigrant and refugee communities on health, health care systems and how to access health care services, how to relate with health care providers	• Allow time and resources to develop health care providers’ competencies
- Lack of social support - Employment Challenges - Transportation - Weather	• Awareness of social support, transportation and employment needs, and challenges of adjusting to poor weather by immigrant and refugee families • Health care and social service providers should liaise with community members to offer support	• Community members to liaise with health care providers and other service providers (e.g. immigration department) to identify and recruit new immigrant and refugee families into the community support network	• Foster relationships with community liaisons

The strength of our qualitative study is that it engaged a large number of African families from 15 different African countries. Families were engaged through multiple interviews conducted in six different languages. Our research team involved members and certified professional translators from different ethno-cultural backgrounds. We were satisfied by the diversity in our sample. On the limitations of our study, we engaged participants from different African countries who might have different factors that affect their experiences of accessing health care in Canada. For instance in some African countries English is one of the official languages thus such participants might have lesser linguistic challenges. However, we did not examine differences in participants experiences based on their countries of origin. Additionally, the cross sectional nature of our qualitative study did not allow us to examine how experiences change over time. Therefore, future work that accounts for our limitations could shed more light on experiences in accessing health care and inform better access programming.

## Conclusion

African immigrant and refugee families face considerable barriers in accessing primary health care services which is reflective of poor social determinants of health. The families in this study experienced challenges because their expectations were not quite met and because of barriers related to facing a new life. In spite of the barriers and poor social determinants of health, both immigrant and refugee families viewed the use of culturally relevant collaborations and networks as opportunities to improve access. In order to improve primary health care access for these groups, policies and programs that seek to address social determinants of health and tap into culturally relevant networking collaborative approaches are needed.

## Abbreviations

DRC: The Democratic Republic of Congo
IFHP: Interim Federal Health Program
SPSS: Statistical Package for the Social Sciences
UN: United Nations
UNHCR: United Nations Refugee Agency
